# How Do Governments’ Policy Priorities Change as the Energy Transition Progresses? A Cross-Country Comparison

**DOI:** 10.1080/13876988.2023.2280270

**Published:** 2023-11-30

**Authors:** Lana Ollier, Marc Melliger, Florence Metz

**Affiliations:** * Research Institute for Sustainability Helmholtz Centre Potsdam, Potsdam, Germany; ** Department of Governance and Technology for Sustainability, University of Twente, Twente, Netherlands

**Keywords:** EU energy policy, comparative analysis, policy analysis, socio-technical transition, sequencing, policy priorities

## Abstract

Today’s energy transition is marked by two key processes: the maturation of the renewable electricity system, and the declining fit between the new and incumbent electricity systems. Given these processes, how do governments change their policy priorities as the transition progresses? Our comparative analysis of six EU member states shows that governments dynamically adapt their policy priorities based on changes in their socio-technical systems. Our initial findings suggest that governments follow a specific sequence of policy priorities in the energy transition. Results stress how important it is that governments strategically sequence policy instruments for a smooth transition amid changing priorities.

## Introduction

A comprehensive reconfiguration of the EU’s energy supply system is required to limit the impact of climate change and achieve net zero emissions by 2050 in alignment with the Paris Agreement. In this transition, climate change mitigation is not policymakers’ sole focus; instead, they pursue multiple policy aims simultaneously, such as maintaining energy security, affordability through limiting costs, and creating competitive domestic industries (Hughes and Lipscy 2013; Schmidt et al. 2019). This inevitably leads to trade-offs between them, where some are given higher priority than others (Sabatier 1988; Hall 1993; Kammermann and Angst 2021). Existing research highlights that governments prioritize these policy aims aligned with pre-existing domestic institutions and beliefs (Sabatier 1988; Ćetković and Buzogány 2016). Yet these priorities may not remain stable during and post-implementation. By creating new “winners and losers”, policy implementation may result in shifting priorities (Meckling et al. 2017).

Technology change plays an important role in (re)defining policy priorities (Schmidt and Sewerin 2017; Edmondson et al. 2019; Schmidt et al. 2019). As new technologies are successfully adopted, learning processes resulting from unforeseen technological and social challenges may lead to shifting policy priorities (Hoppmann et al. 2014). We therefore explore the interplay between governments’ policy priorities and socio-technical systems (STS). STS consider the “linkages between elements necessary to fulfill societal functions” (Geels et al. 2016). The concept highlights the interconnection between social and technical elements, whereby the reconfiguration of STS is an incremental process.

We explore whether governments sequence their policy priorities as the energy transition progresses. Previous research has shown that low-carbon leaders employ a distinct sequence of specific policy instruments (Meckling et al. 2017). The research on policy sequencing in the climate and energy policy field shows that instrument choices strongly interact with the STS transition (Leipprand et al. 2020). It remains unclear whether a similar relationship exists between general policy aims and STS properties. High-level policy aims differ from specific instrument choices in that the former are typically considered relatively stable and also influential in shaping policy instrument decisions (Hall 1993).

This exploratory study asks whether certain STS properties are characteristically associated with governments’ policy priorities in different phases of the energy transition. We understand STS properties here as the way in which social and technical system features are organized and interconnected. We explore patterns in the parallel evolution of renewable electricity (RE) STS properties and governments’ policy priorities as different and new policy problems emerge in various phases of the transition. Governments’ policy priorities are shaped by the interaction of various actor groups (Lauber and Schenner 2011; Boasson 2020; Meckling and Nahm 2022; Andersen et al. 2023). Changing priorities can therefore be traced back to specific actor coalitions and their influence on the policymaking process. When the preferences or power dynamics of involved actors change due to the transition (Jacobsson and Lauber 2006), governments’ priorities also shift over time (Hall 1993). In this paper, we focus on identifying broader patterns of interaction between STS properties and government priorities. The identified patterns also provide additional insight into why countries dismantle or expand their policy schemes. To this end, we compare six EU countries: France, Germany, Italy, Spain, Sweden, and the UK, all of which have a long history of promoting climate and energy policy, but nevertheless differ in their progression in the energy transition.

The paper is structured as follows: First, we provide an overview of our sample countries’ transition status and their corresponding support schemes. Next, we conceptualize STS properties and policy priorities, and formulate expectations around their interaction in different stages of the energy transition. Subsequently, we outline our data analysis approach, which involves examining a combination of quantitative and qualitative data to identify patterns of interaction. We then discuss our findings on the relationship between STS properties and policy priorities before drawing conclusions on both results and methods employed.

## The Sample: Contextualizing EU Member States’ Policy Priorities and Transition Progress

Our sampled EU countries^1^ have a similarly lengthy history in supporting RE development through designated yet different support schemes, with some experiencing more drastic reforms than others. Despite their early efforts to support RE development, their transitions have progressed in differing ways (for more detail see Online Appendix Table B2).

In our sample, Sweden is the most advanced in its transition, with over 50 per cent RE share in the early 2000s, which has been growing gradually since. Previous research shows that a core element of the country’s transition was to strike a balance between climate measures and economic efficiency, aiming to ensure deployment of renewables at the lowest cost to society as a whole (Bergek and Jacobsson 2010). This was achieved through the implementation of a green certificate trading scheme.^2^

Spain has also progressed relatively far in its transition, but lags behind Sweden. The country started with a relatively high RE share in the early 2000s (around 19 per cent) and continued on an average growth trajectory. One main driver behind the transition in Spain had been the aim of establishing a domestic market for renewable energy (Lauber and Schenner 2011; Haas 2017). To protect and grow domestic industries, Spain used a market-pull, tariff-based instrument.^3^ In 2004, the government introduced a feed-in tariff (FIT)^4^ that was amended several times and ultimately dismantled in 2012. In 2014, a new tender-based support scheme was established.

Italy has followed a similar trajectory. The country began with a slightly lower RE share than Spain, around 16 per cent, in the early 2000s, with relatively modest growth until around 2010, when the deployment of renewables picked up speed. Italy supported RE deployment through a FIT, which was introduced in 1992, and a quota system (green certificates), established in 1999 (Prontera 2021). The scheme was amended in 2005 and 2007. In 2012, the government introduced competitive auctions for larger installations alongside the FIT scheme for small and medium-sized installations (Prontera 2021).

France, despite having a similar starting point to Italy in the early 2000s (13 per cent share), experienced extremely modest growth. In 1996, the country established a tender-based feed-in premium^5^ to explore wind energy (Laali and Benard 1999). In 2000, France introduced a technology-specific FIT aiming to develop the domestic renewables industry, which was subsequently reduced twice in 2010 due to cost concerns. In 2015, the French government introduced technology-specific feed-in premiums for larger projects and fixed feed-in tariffs for small-scale renewables (CEER 2018; Boasson et al. 2020). The success of these support schemes was limited, and by 2014 the share of renewables was still below 15 per cent.

For other countries, the effective STS transition only started later. Germany (around 9 per cent) and the UK (around 3 per cent) had a relatively low RE share until 2010 but continued on a very strong growth trajectory (Mitchell and Connor 2004). Nonetheless, both countries had very different support schemes to unlock growth. Taking a similar approach to Spain, Germany adopted its first comprehensive FIT in 2000, which was amended several times thereafter (Cox and Dekanozishvili 2015). In 2012, a market premium was introduced alongside the FIT to advance market integration. Like Sweden, the UK initially adopted a green certificates scheme in 2002. A decade later, this scheme was substituted and then replaced by Contracts for Difference^6^ for large-scale projects and a FIT for small-scale generation (Rayner et al. 2020).

The six countries, despite being early adopters, have experienced different STS development. Even when countries adopted similar support schemes, they followed different growth trajectories. The varying progress in their RE STS transitions allows us to observe how countries differ in the way they typically sequenced their policy priorities.

## Conceptual Framework: Policy Priorities and STS Properties

STS properties may drive changes in governments’ policy priorities at different stages of the transition. Cashore and Howlett’s taxonomy of policy elements differentiates between policy “ends” and “means”. “Ends” describe the specific aims that the policy wants to achieve (we therefore refer to them as “aims”), while “means” capture the tools to reach the aim (Cashore and Howlett 2007). Much of the existing research analyzes policy change from the perspective of the “means” (Meckling 2019; Lockwood 2022). Conversely, we look at policy priorities in terms of high-level “aims”, whose changes are infrequent but which have been recorded for climate and energy policy in various contexts (Kern et al. 2014; Schmidt et al. 2019). Aims include both policy goals and more concrete objectives. Examples of goals include maintaining affordability or energy security supply, reducing environmental and climate impact, and increasing domestic industry competitiveness (Hughes and Lipscy 2013; Schmidt et al. 2019). These goals are reflected in more operational objectives, such as enhancing energy efficiency, expanding renewable energy supply, and fostering system flexibility (Markard 2018; Ollier et al. 2020).

We capture the different stages of the transition here through the lens of STS. The concept highlights the co-evolution and interconnection of technology and society. As they transition, STS exhibit distinct properties in different stages of the transition. The transition of STS can typically be characterized by two key properties.

First, a successful transition relies on market formation for new technologies. This is especially relevant in the context of decarbonization, where the market needs to provide low-carbon alternatives for existing technologies (Hekkert et al. 2007). In this process, social functions develop alongside the technology (Bergek et al. 2008). In the STS’s early stages, technologies are still immature and the prevalent dynamics comprise learning and exploration (Jacobsson and Lauber 2006; Bergek et al. 2008). Technologies are generally not profitable and are heavily reliant on financial support from governments. During the process of maturation, market-reinforcing benefits materialize as technologies shift from exploration towards exploitation and gaining market share. As technologies are implemented on a larger scale, the total cost of government support schemes may increase, even if the costs per unit decline (Lauber and Jacobsson 2016). Mounting public costs can then place pressure on policymakers to decrease government support and create more cost-efficient policies (Edmondson et al. 2019). In this context, political pressure arises from those who bear the costs. This can include consumers, energy-intensive industries, and energy utilities (Leiren and Reimer 2018; Meckling et al. 2022). Upon reform, pressure on policymakers to reduce costs relaxes (Pahle et al. 2018). Ultimately, when technologies become fully mature, they constitute the dominant design (Bergek et al. 2008).

In this context, our expectation is that countries experiencing a phase of high RE STS maturation are most likely to prioritize cost limitations as a policy aim. Costs are understood here as economic costs, which include electricity prices and policy costs associated with support schemes. Hence, our first expectation (E1) is that: *With a high increase in RE STS maturation, governments increasingly prioritize limiting policy cost as a policy aim.*

Secondly, the transition to low-carbon technologies, specifically renewables, involves the interaction of the new with the incumbent system, whereas the latter locks in existing technology (Geels et al. 2016). While new STS initially emerge as a niche within the existing system at the early stages of a transition, their maturation and institutionalization brings about a growing potential to challenge the established system (Ford and Newell 2021).

The interaction between the new and incumbent systems is a key dynamic within a transitioning STS. In this context, socio-technical fit is a key property of the STS. Socio-technical fit can be understood as the fit of a new technology within an existing STS. In our specific case, one prevalent characteristic of the existing electricity system is that it “evolved with dispatchable energy sources that could respond to changes in demand, thus serving customers at any time” (Smith 2020). Renewable electricity does not share this feature of flexible supply. The energy system therefore needs to undergo a transition from a system with a stable supply that meets baseload supply through thermal plants at all times, to a system with increasing amounts of variable supply through RE (Smith 2020; Ollier et al. 2022). In this context, the socio-technical fit describes how well RE can be integrated within the existing STS, while maintaining stable supply.

In the early stages of this transition, a dominant amount of dispatchable energy remains. The “socio-technical fit” decreases with a growing amount of intermittent electricity. To overcome this socio-technical misfit, governments can increase their electricity system’s flexibility through options like demand-side management, storage, or cross-border transmission (Tröndle et al. 2020; Thonig et al. 2021). System flexibility, in general, is the ability to adjust supply and demand to maintain stability of the system. In this situation, we anticipate countries with a higher share of intermittent RE but limited means to balance supply (for example through cross-border trade) to prioritize system flexibility as a policy aim. Political pressure may be driven by system operators and regulators with responsibilities for system maintenance, which are both dependent on a high socio-technical fit in providing their services (Smith 2020). Consequently, our second expectation (E2) is that: *With a decreasing socio-technical fit of the new and incumbent energy systems, governments increasingly prioritize system flexibility as a policy aim.*

These dynamics emphasize how STS properties evolve throughout different stages of the energy transition. Governments may not encounter challenges simultaneously, but they are likely to confront similar obstacles as they transition their energy system.

## Data and Data Analysis

For our sample of six countries, we analyze the time period between the adoption of the first RE support schemes in the early 2000s and the late 2010s. Comparing early policy priorities with those in the late 2010s, we gain insight into the ways governments adapt their priorities in line with their changing RE STS. We build here on the assumption that there exists a time lag between the STS change and the corresponding shift in priorities.

Our analysis is a plausibility probe^7^ of a characteristic relationship between STS properties and policy priorities in different stages of the transition. To test the relationships as formulated in E1 and E2, we analyze patterns of interaction between STS properties, i.e. levels of maturity increase and socio-technical fit, and changes in policy priorities in a cross-country comparison.

We measure the increase in RE STS maturity and socio-technical fit using a set of quantitative indicators (for more detail see Online Appendix Table A1). To capture STS maturity increase, we measure changes in (1) RE growth rate, (2) employment in the RE sector, (3) RE support in EUR/MWh, and (4) levelized cost of electricity (LCOE). We measure changes in most of these variables between 2010 and 2014 to capture policy-driven STS change. However, for the RE growth rate, we need a longer time period to observe a trend, so we measure it from 2004 onwards. We consider a country to have a high increase in maturation if it exhibits a high RE growth, a high increase in employment, a high increase in support, and a high decline in LCOE over time. Using these indicators, we determine the increase in maturity relatively, by categorizing countries as having either a low or high increase in maturity relative to the other countries in our sample. Similarly, we determine higher/lower fit relatively, by comparing the value of each socio-technical fit indicator to the average in our sample. To determine socio-technical fit as an increasing amount of variable energy with limited flexibility options, we measure (1) the 2014 share of RE supply, (2) changes in the electricity wholesale price, and (3) cross-border transmission capacity. These indicators capture both the share of intermitted electricity and the ability to balance supply, assuming that RE brings down wholesale prices in times of high supply. We consider a country to have a high socio-technical fit if it meets the following criteria: low RE share, a high cross-border transmission capacity, and a low decrease in electricity price (see Online Appendix B for further details for criteria used in this classification). For both variables, we classify countries where they meet the most criteria.

We consider increasing or decreasing policy prioritization as the change in relative importance of a policy aim over time in comparison to other policy aims. To capture policy priorities, we manually coded policy aims from governments’ political strategy documents^8^ (see Online Appendix Table B1). Political strategies are formal government documents, which lay out a long-term plan for a specific policy area. These documents define policy goals and serve as a guide or blueprint for the government’s actions and decisions. Selected documents are coded using content analysis. Our codebook uses Cashore and Howlett’s taxonomy of policy elements, which differentiates between goals and objectives for policy aims (Cashore and Howlett 2007). Out of the resulting seven coding categories, we focus our analysis on two policy aims, namely “cost limitations” and “system flexibility” (see Online Appendix Table A2). We use the other coded policy aims for comparison, i.e. to analyze whether governments have prioritized cost limitations or system flexibility, respectively, more (or less) than other policy aims. During the coding process, we use specific cue statements to identify support for these policy aims. For instance, regarding system flexibility, we look for cues indicating the importance of demand- and supply-side measures, infrastructure extensions, and smart grids.

The priority given to a policy aim is represented by the number of times the policy aim was expressed relative to other aims (in percentages). To determine increasing or decreasing prioritization, we need to compare two points in time (TP). Hence, we choose a political strategy published in every country in the early 2000s (hereafter TP1) and one towards the end of the 2010s (hereafter TP2) as points of comparison. We assess increasing or decreasing prioritization as percentage increase or decrease of the relative importance between TP1 and TP2. Where all governments follow the same trend in prioritizing a policy aim, we compare the individual increase to the average increase of this government’s other policy aims to classify it as relatively higher/lower prioritization.

We analyze patterns of interaction between STS properties and policy priorities in a two-by-two typology: one dimension captures STS changes (high/low increase in STS maturity and high/low socio-technical fit), and the other measures changes in policy prioritization (high/low (de-)/prioritization). In this typology, countries are categorized regarding their expression of both variables. We contextualize the identified patterns by taking into consideration countries’ varying transition stages.

## Results

### Technology Maturation and Cost Limitations

**Figure 1.**
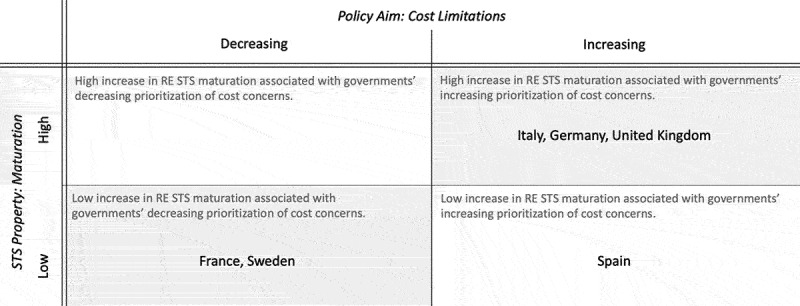
Typology of changing government priorities in the RE STS transition: Relationship between RE STS maturation and the policy aim of limiting costs (for underlying data see Online Appendix Table A3/B2). The boxes in grey support our expectation E1.

When examining the specific patterns of interactions between prioritizing cost limitations and STS maturation in Figure 1, countries can be categorized into two different groups, with Spain occupying its own category (for underlying data see Online Appendix Table A3/B2).

The first group (top right corner of Figure 1) consists of countries with high maturation that prioritize cost limitations. Italy, Germany, and the UK fit here. Although at differing stages of their respective transitions, all three countries have fairly dynamic STS, where high maturation constitutes one key characteristic. In all three cases, support levels increased alongside growing market shares. Despite a lower-than-average support level (+1.74), we find the highest growth rate in the UK, where RE grew more than 500-fold from 2004. The UK is also the only country showing significant growth in RE employment and a reduction in the LCOE (0.14 USD/kWh). The decline in LCOE here is explained by reductions in supporting material costs and cost of labor,^9^ indicating growing efficiency and learning effects for both. The UK’s substantial RE growth-and-employment rate can be partially attributed to its less developed STS in the early 2000s (Mitchell and Connor 2004). Nevertheless, it also indicates a phase of increased maturation, which corresponds with an increasing prioritization of cost limitations as a policy aim (+19 per cent). It is worth noting that the UK government prioritizes cost limitations despite its comparatively low increase in support levels.

In contrast, both Germany (+4 EUR/MWh) and Italy (+10 EUR/MWh) experience a substantial increase in support levels. In Germany, this corresponds with a very high RE growth rate (298 per cent), which is lower in Italy (207 per cent). In terms of cost development, the LCOE declines more in Italy (−0.13 USD/kWh) than in Germany (−0.1 USD/kWh). Employment is nearly stable in Germany (−3 per cent), but significantly declines in Italy (−38 per cent), likely due to the impact of the 2010 euro crisis on Italy’s general economy. Although the economic crisis has a greater impact on Italy, the policy priority of cost limitations rises more steeply in Germany (+17 per cent) than in Italy (+8 per cent), thereby suggesting that the crisis may not be the primary driving factor behind policy prioritization in these cases.

All three cases support our first expectation. They highlight the plausibility for governments in a phase of high RE STS maturation to prioritize cost limitations as a policy aim. The UK’s case also indicates that cost concerns might be decoupled from support levels.

The second group (lower left corner), which includes France and Sweden, is characterized by a low maturation and de-prioritization of cost limitations. This finding is intriguing as Sweden is the furthest progressed and France the least progressed in their transition. A detailed analysis of these cases reveals their commonalities and differences, shedding further light on why both governments de-prioritize cost limitations (FR = −11 per cent, SE = −30 per cent). The two countries’ RE growth rate is almost identical (FR = 133 per cent and SE = 123 per cent), but the reasons for this comparatively low growth rate are different. Sweden’s lower growth rate results from its already high share of renewables, while France simply has low RE deployment. This is also evident from the other maturity indicators. Sweden’s RE support levels are slightly decreasing (−0.2 EUR/MWh) and both the LCOE (almost 0 USD/kWH) and employment remain relatively stable (−8 per cent). On the other hand, France has nearly stable support levels (+0.1 EUR/MWh) and stable job numbers (−4 per cent), but a continuing downward LCOE trend. The decreasing LCOE indicates that technical improvements and learning are ongoing.

In summary, Sweden de-prioritizes cost concerns as it decreases government support in a relatively mature system. By contrast, in France, the low level of concern about costs is more likely to be the result of a slow RE uptake. These cases illustrate that low maturation, alongside decreasing prioritization of cost limitations, can occur at different stages of the transition. Both cases in this category support our expectation that governments prioritize cost limitations in a fast-maturing STS, as they also demonstrate the opposite: governments de-prioritize cost limitations in slow-maturing STS.

In our sample, Spain takes an outsider position as the government prioritizes cost limitations (+6 per cent) despite showing signs of a slowing maturation. This trajectory is evidenced by a below-average growth rate (+199 per cent), declining support levels (−0.2 EUR/MWh), a stable LCOE trend (−0.09 kWH), and declining employment (−38 per cent). It is essential, however, to note that due to a lack of data,^10^ we are examining Spain during the midst of the economic crisis, which further emphasizes cost concerns (Gürtler et al. 2019). Still, this case contradicts our first expectation (E1) since the country exhibits an increasing prioritization of cost limitations despite only a minimal increase in RE STS maturation.

To conclude, five out of six cases support our first expectation (E1). Our results illustrate that prioritizing cost limitations is most prevalent in rapidly maturing RE STS. Hence, in this case, our expectation withstands the plausibility probe, as the identified pattern implies an association between STS property and policy priority.

### Socio-technical Fit and System Flexibility

**Figure 2.**
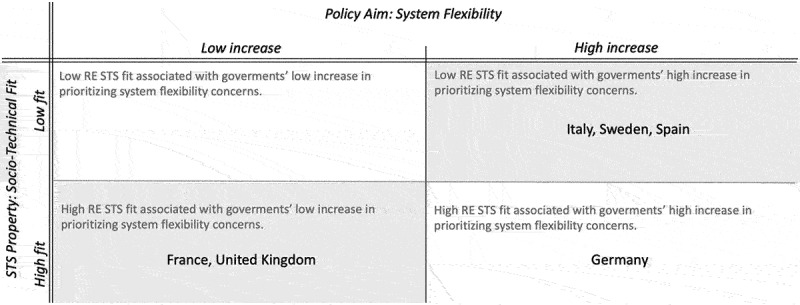
Typology of changing government priorities in the RE STS transition: Relationship between socio-technical fit of the new and incumbent electricity system and the policy aim of system flexibility (for underlying data see Online Appendix Table A3/B3). The boxes in grey support our expectation E1.

In our sample, all governments demonstrate an increasing prioritization of system flexibility. Therefore, our typology in Figure 2 displays varying degrees of prioritization rather than de-prioritization. When analyzing patterns of interactions between the prioritization of system flexibility and socio-technical fit, countries can be classified into two groups, with Germany forming its own distinct category (for underlying data see Online Appendix Table A3/B3).

Our typology’s first group (top right corner) is formed by Italy, Sweden, and Spain, which show a low socio-technical fit while simultaneously prioritizing system flexibility concerns. More detailed analysis of these countries highlights variations in socio-technical fit, which align with their level of prioritizing system flexibility. These countries all have in common that they are relatively further advanced in their transition concerning RE shares (SE = 62 per cent, ES = 40 per cent, IT = 37 per cent). At the same time, they have some of the lowest cross-border transmission capacities, which limits their ability to balance supply across borders (SE = 10 per cent, ES = 24 per cent, IT = 25 per cent). Nevertheless, negative price development resulting from sudden increases in (variable) renewables supply is observed only in Sweden and Italy (SE = −29 EUR/MWh, IT = −16 EUR/MWh). This difference in socio-technical fit is reflected in the two countries’ higher priorities for system flexibility (SE = 31 per cent, IT = +30 per cent) compared to those of Spain (+14 per cent). These findings create support for our second expectation (E2). From these results, it is plausible that a high prioritization of system flexibility is characteristic for countries with a low socio-technical fit. In fact, the lower the fit, the higher the prioritization.

The second group (lower left corner), which includes the UK and France, further supports this finding. Indicative of their high socio-technical fit, RE is still relatively little developed in both countries (both 18 per cent share). However, further analysis of their socio-technical fit shows that both countries have reasons, although different, to be concerned about a declining fit, which can explain their growing system flexibility concerns. The UK has the lowest transmission capacity in our sample (5 per cent), putting the currently positive price development (+1 per cent) at risk of becoming negative due to a lack of ability to balance supply across borders. In France, despite the country’s better ability to balance supply (28 per cent transmission capacity), handling high RE injections already proves challenging and results in negative price development (−16 EUR/MWh). This is evident in the growing concern for system flexibility in both countries (FR = +7 per cent, UK = +11 per cent), though not to the same extent as in the first group. These cases further support our second expectation (E2) by demonstrating that low prioritization of system flexibility can be attributed to a higher socio-technical fit. Nonetheless, we are already witnessing system flexibility becoming a more central concern as the share of RE grows.

Germany stands out as an outlier in our typology, falling into a category characterized by a high socio-technical fit and high prioritization of system flexibility as a policy aim. It exhibits several features of a high socio-technical fit, such as a relatively low share in RE in 2014 (27 per cent) and a high transmission capacity (68 per cent). Nonetheless, system flexibility as a political priority is rising steeply over our period of observation. While part of this trend may be explained by the country’s negative electricity price development (−14 EUR/MWh), a lowering socio-technical fit cannot fully account for this steep rise in flexibility concerns. Therefore, this case does not create support for our second expectation (E2).

In conclusion, five out of six cases support our second expectation (E2). The results indicate that a prioritization of the policy aim system flexibility is most prevalent in countries that are characterized by a low socio-technical fit. This pattern further reinforces our claim of an association between STS properties and policy priorities.

## Discussion

Our plausibility probe generally supports the claim that specific policy priorities are characteristically associated with specific STS properties. Therefore, our findings provide a starting point for future research to explore this relationship in more detail.

While STS properties associated with a specific transition stage plausibly explain policy prioritization in five out of the six cases for each expectation examined in this study, the fact that Germany and Spain remain outliers also reveals the limitations of our research. On the one hand, aside from STS properties, the larger political and macroeconomic context plays an important role in defining policy priorities. Notably, the initial expansion of renewables occurred amidst economic and political crises, with Southern European countries being particularly affected. On the other hand, however, there are variations in data coverage for policy aims. As Spain has not published a strategy since 2011, changing policy priorities resulting from STS change may not be fully captured here. Despite their limitations, strategy documents offer a unique data source in understanding and comparing high-level policy aims.

When replicating this exploratory study across a larger set of countries, it is essential to keep in mind that government priorities coded from strategy documents may be considered somewhat of a “black box” as they ignore the underlying politics. Nonetheless, we believe that our approach is practical for capturing and comparing governments’ policy priorities. Additionally, the indicator set for STS properties may not fully capture STS maturation and socio-technical fit. Nevertheless, we believe our indicators effectively capture the main trends in this plausibility probe. In the early stages of transition, we found LCOE and employment trends particularly important indicators for understanding maturation increases. However, their predictive value diminished in later stages. We also found that all three indicators of system flexibility provided important contextual information about countries’ transition stage, yet negative wholesale prices seem to have been key in placing the issue on political agendas. We encourage further quantitative research to validate these indicators.

When generalizing beyond our cases, it is crucial to emphasize that the selected countries in our study all actively promote RE expansion and are experiencing effective STS change, albeit to varying degrees. Such efforts are not uniformly observed across or beyond the EU, where renewables expansion is progressing at highly variable rates. In this context, our study does not address the question of a “minimum” STS change required to influence policy priorities.

## Conclusion

This study combines transition research and public policy analysis to explore how governments’ policy priorities change as the energy transition progresses. Thereby, we provide insights into transition dynamics towards stable transition pathways.

In the context of the transition literature, our findings contribute to previous research on policy sequencing and policy change more broadly (Meckling et al. 2017). We add to this literature in several ways: First, by detailing an important driver of the evolution of policy instruments: STS properties. Our analysis illustrates that changing RE STS properties bring forth distinct policy priorities, which affect choices of policy instruments. Hence, evolving policy aims may serve as an important mediating factor between RE STS change and instrumental sequencing throughout the transition process. Specifically, mounting cost concerns emerging at the end of the first decade of the 2000s in Germany, Italy, the UK, and Spain paved the way for subsequent policy reform in favor of tender-based feed-in premiums.

Second, our research indicates that countries in a transitioning RE STS follow a distinct sequence of priorities. While we suggest more systematic research on this, our results present some initial findings on such a policy priority sequence driven by STS properties. Concretely, they indicate that cost concerns start off low in the initial stages of the RE STS but increase as the system expands. As growth plateaus, however, other priorities take precedence. One such priority is system flexibility, which becomes more relevant in countries with limited capacity to balance energy supply, especially in the later transition stages.

The identified sequence of priorities can be an important driver for countries to dismantle or expand their RE support schemes. As priorities change, support policies often do too. Whereas all countries in our case study eventually prioritized cost concerns, the level of change to their RE support schemes varied: Spain completely dismantled its support scheme, Italy and the UK made substantial changes, while Sweden, Germany, and France made more incremental adjustments. These incremental adjustments created a more stable policy environment for STS stakeholders than the Spanish policy dismantlement. To create a stable policy environment in transitions, governments could anticipate reform needs by planning ahead and strategically sequencing support schemes in accordance with changing priorities (Pahle et al. 2018).

Competing ideas and priorities regarding the transition pose a major barrier to European coordination and ambitious climate policies. Yet our results indicate that the transition might foster greater policy alignment. The concept of a sequence of priorities suggests that as countries progress in their transition, their policy priorities may align more closely as they encounter similar challenges in their progressive transition. Similar priorities could then form a basis for more ambitious climate policy. However, this assumption relies on the effective transformation of STS and may be more applicable to later-stage transition dynamics.

Building on this plausibility probe, using a larger set of cases, we invite further research on the association between STS properties and policy priorities, and the ways these can facilitate policy convergence beyond the specific context of the EU. For instance, the United States could provide an interesting case, because states have historically been the main driving forces behind climate and energy policy (Martin and Saikawa 2017) and are in differing stages of their respective transitions.

Further research is required to understand how specific STS properties influence governments’ prioritization of policy aims. Our initial findings illustrate the specific challenges governments encounter during various stages of the transition to renewable electricity systems and policymakers’ strategic responses to these challenges. Moreover, our process-oriented understanding of transitions highlights the importance of strategic policy instrument sequencing to enable a smooth transition amid changing government priorities.

## Supplementary Material

Supplemental Material

## Notes

1. During our analysis, the UK was part of the EU.

2. Suppliers must purchase tradable green certificates corresponding to a RE quota, which are then traded in a separate market.

3. Market-pull instruments increase demand for RE by offering economic incentives through tariff-based mechanisms.

4. Feed-in tariffs offer long-term contracts to RE producers above market price.

5. Uses a competitive tender process to award contracts for RE production, producers sell to the electricity market and receive a premium on top of the market price.

6. Long-term contracts, allocated through tenders, between a generator and the Low Carbon Contracts Company where deviations from a reference price are paid out to the contractual partners.

7. A plausibility probe is a preliminary exploratory study, often using a case study, to test the credibility of a hypothesis or theory

8. In 2012, Spain changed its climate and energy policy, ending support for renewable energy. This change happened during our analysis period, when Spain didn’t publish a strategy document. We used government decree RD 1/2012, issued as an emergency measure, to reflect this shift by the conservative government, aimed at stopping the tariff deficit’s growth.

9. In the LCOE data we use, differences are mainly attributed to regional variations in supporting material and labor costs, while an average cost of capital is assumed (see also Online Appendix Table B1).

10. Spain dismantled its support for renewable energy in 2012 and did not establish a new strategy until 2021. Instead of a comprehensive strategy, we coded government decree RD 1/2012, which predates the other documents coded for TP2.
