# Supplementary material for: How Do Governments’ Policy Priorities Change as the Energy Transition Progresses? A Cross-Country Comparison
Source: J Comp Policy Anal. 2023 Nov 30;26(3-4):251–65. doi: 10.1080/13876988.2023.2280270 (PMC11318110; doi:10.1080/13876988.2023.2280270)
Supplement: Supplemental Material [file FCPA_A_2280270_SM2720.docx]

**Supplementary Material**

**Appendix A: Data and data analysis policy priorities**

**Document selection**

In capturing policy priority change, we manually code policy aims from strategy documents (including a white paper in the UK^[[1]](#footnote-1)^). As governments publish such strategies regularly and in a comparable format, they provide a sound basis to gather data on priority changes. We select strategy documents that hold at least one of the following keywords in their title: “Renewable energy/electricity,” “Energy/electricity,” “Energy Transition,” “Climate”, “Decarbonis-ing/-ation”, “Carbon,”, “Greenhouse gas” and “Sustainability”. We exclude draft bills or draft strategies. We are interested in changes that occurred between adoption of initial strategies in the early 2000s, which shaped the implementation of initial support schemes, and strategies published in the later 2010s, where we would expect to see an impact from STS change. As the publication dates of the political strategies are not identical across countries, we choose the last strategies published before the cut-off dates in 2000 (TP1) and 2018 (TP2). There is one notable exception to this. In 2012, Spain departed from its climate and energy policy strategy in abandoning all RE support. As this drastic shift occurred within our time period of analysis, we add the government decree RD 1/2012, which issued as an emergency battery of measures to stop the growth of the tariff deficit and reflect the strategic change of conservative government, to our list of coded political strategies. The document selection for each case is discussed between the coders to ensure coherence. For a list of all coded documents, see Table A1.

**Table A1**: List of all coded documents

| Country | Year | Name | TP |
| --- | --- | --- | --- |
| Germany | 2007 | Eckpunkte für ein integriertes Energie- und Klimaprogramm | TP1 |
| United Kingdom | 2007 | White Paper on Energy – Meeting the energy challenge” | TP1 |
| Italy | 2002 | Piano nazionale per la riduzione delle emissioni di gas responsabili dell’effetto serra – 2003-2010 | TP1 |
| Spain | 2007 | Estrategies Espanola de Cambio Climatico y Energia Limpia Horizonte 2007-2012-2020 | TP1 |
| France | 2004 | Plan Climat | TP1 |
| Sweden | 2002 | Samverkan för en trygg, effektiv och miljövänlig energiförsörjning | TP1 |
| Germany | 2017 | Strom 2030 Langfristige Trends – Aufgaben für die kommenden Jahre | TP2 |
| United Kingdom | 2017 | The Clean Growth Strategy: leading the way into a low carbon future | TP2 |
| Italy | 2017 | Strategia Energetica Nazionale | TP2 |
| Spain | 2012 | Real Decreto-ley 01/2012 | TP2 |
| Spain | 2011 | Plan de Energias Renovables 2011-2020 | TP2 |
| France | 2018 | Stratégie Nationale Bas-Carbone | TP2 |
| Sweden | 2018 | Energipolitikens inriktning | TP2 |

**Content analysis**

All documents are manually coded in their respective national language using content analysis. The strategies are coded by four researchers, all knowledgeable on EU and national energy policies in the countries they coded. During the coding process, each coder examines every sentence individually and decides a) whether it reflects a policy aim and b) to which category in the coding scheme it belongs. The policy aims are coded every time they occurred throughout a document. To increase inter-coder reliability, coders analyzed one text in common and discussed differences in the outcome where they occurred. This process was repeated with different texts until coded samples showed a high level of similarity (around 80%). Using the Discourse Network Analyzer^[[2]](#footnote-2)^, we collected 4666 statements across 13 strategy documents.

**Codebook**

The codebook is predetermined (top-down) based on Cashore and Howlett’s taxonomy of variable policy elements (Cashore and Howlett 2007), which provides us with a structured frame to compare policy aims at the same level of abstraction. Our analysis focuses on “Cost Limitations” and “System flexibility” (grey in Table A2). As we propose that these become relatively more important compared to other policy aims, we analyze all policy aims in our coding scheme to determine relative importance to one another. To understand how the individual policy aims were prioritized, we employ frequency analysis. We first created an affiliation matrix from our coded data that displays the strength of each policy aims (in columns) for each country (in rows). The strength of a policy aim is represented by the number of times a policy aim was mentioned relative to other policy aims (in %). This accounts for countries that published longer strategy documents.

**Table A2:** *Coding scheme policy aims in RE policy.*

| **Policy Aims** | |
| --- | --- |
| **Energy System Goals** | **Energy System Objectives** |
| **Cost limitations,** expressed by one of the following cue statements referring to economic costs:   - Importance of limiting costs - Concerns of cost of policy measures - Concerns of cost of electricity generation (over time) - Concerns about financial feasibility - Concern about the price of electricity | **Increased system flexibility,** expressed by one of the following cue statements:   - Importance of demand-side flexibility (measures) - Importance of supply side-flexibility (measures) - Importance of grid/infrastructure extension - Smart grid |
| **Increased supply security**, expressed by one of the following cue statements:   - Importance of supply security - Electricity independence - Concerns on declining indigenous supply (fossil fuels) | **Increased renewables**, expressed by one of the following cue statements:   - Relevance of renewable electricity generation - Importance of renewables support schemes - Ambitious renewable energy targets |
| Protection of **climate/environment**, expressed by one of the following cue statements:   - Importance of climate and environmental protection - Comprehensive (international) climate goals - Climate leadership | **Increased decarbonization**, expressed by one of the following cue statements:   - importance of decarbonization of the electricity supply system - Importance of ambitious greenhouse gas reduction targets |
| **Increased economic benefits**, expressed by one of the following cue statements:   - Industrial policy, long term economic benefits - Benefits of technology development and innovation |  |

Note: The policy aims in the row highlighted in gray is the variable we will analyze in line with our expectations; the other policy aims we use as a means for benchmarking the relative importance of these policy aims.

**Data Analysis**

To compare policy priority changes across countries and time, we first measure the priority changes between TP1 and TP2 as percent increase or decrease. This provides us with an indication whether countries prioritize or de-prioritize individual policy aims. If all countries follow the same trend, it may become necessary to further classify this change. In these cases, we compare the individual increase/decrease to the country’s average increase/decrease to classify it as high/low increase/decrease. We present the complete analysis of all policy aims in the Table A3.

**Results policy preferences**

**Table A3***: Results of the analysis*

| Country | Policy aim | TP 2 percent | TP 1 percent | Policy Preference change | Group |
| --- | --- | --- | --- | --- | --- |
| ES | Cost limitations | 16% | 11% | 5% | Prioritization |
| DE | Cost limitations | 26% | 9% | 17% | Prioritization |
| SE | Cost limitations | 19% | 49% | -30% | De-prioritization |
| UK | Cost limitations | 30% | 14% | 16% | Prioritization |
| IT | Cost limitations | 8% | 0% | 8% | Prioritization |
| FR | Cost limitations | 6% | 14% | -11% | De-prioritization |
| ES | System. Flex. | 22% | 8% | 14% | Prioritization (high) |
| DE | System. Flex. | 45% | 21% | 36% | Prioritization (high) |
| SE | System. Flex. | 41% | 11% | 31% | Prioritization (high) |
| UK | System. Flex. | 30% | 14% | 11% | Prioritization (low) |
| IT | System. Flex. | 30% | 0% | 30% | Prioritization (high) |
| FR | System. Flex. | 9% | 1% | 7% | Prioritization (low) |

Source: Own data

**Annex B: Data and data analysis: socio-technical system properties**

**Data**

In our study we focus on two distinct STS properties: increase in RE STS maturation and socio-technical fit of the new and incumbent energy systems. To understand changes in these variables we gather quantitative data (summarized in Table B1). Generally, our goal is to assess these variables between TP1 and TP2. Hence, we specifically chose the time period from 2010 to 2014 for our analysis

**Table B1:** *Details and data sources for the indicator set*

| **Expectation** | **Variable** | **Indicator** | **Details on calculation method** | **Data Source** |
| --- | --- | --- | --- | --- |
| E1 | Increase in technology maturity | Increase/decrease EUR/MWh RE support between 2010 and 2014 | We multiply the per unit support with the size of the RE support scheme. Per unit (MWh) support in EUR is calculated for all new installations under different support schemes (FIT, Investment Grant, Green Certificates, FIP, Call for tender). The size of the RE support scheme is measures as the total gross electricity receiving support relative to the total electricity produced. This includes support for landfill gas, biogas, hydropower, PV, wind on and offshore, solid biomass, geothermal, ocean and CHP. | Council of European Energy Regulators status (CEER) reports in 2011 and 2015 (CEER 2011, 2015) |
|  |  | Growth/decline of the average (onshore wind projects/PV) levelized cost of electricity (LCOE) in USD/kWh between 2010 and 2014 | The LCOE of a given technology is the ratio of lifetime costs to lifetime electricity generation, both of which are discounted back to a common year. We use the LCOE for wind and solar, provided by IRENA. IRENA’s LCOE is based on the total cost of completing a project (e.g. including project development costs, grid connection, equipment, installation, civil engineering, contingency, etc.) and capacity factors, calculated as the ratio of annual generation relative to the theoretical continuous. It does not include the specific costs of capital where IRENA uses an average. Differences in LCOE are thus predominantly explained by regional variations in supporting material costs and cost of labor. Our study uses the average LCOE increase/decrease for onshore wind and solar, as these are the two dominant sources of RE in the EU. | IRENA (IRENA 2020) |
|  |  | RE growth rate between 2004 and 2014 in % (2004) | Growth in the rate of renewable energy in gross electricity consumption indexed from 2004. | 11^th^ EurObserv’ER Report (EurObserv’ER 2011), 15^th^ EurObserv’ER Report (EurObservER 2015) |
|  |  | Growth/decline in direct and indirect jobs in RE sectors between 2010 and 2014 in % | Direct and indirect jobs in the following sectors: wind, biomass, photovoltaic, biofuels, heat pump, biogas, hydro, solar thermal + CSP, geothermal and waste. Indirect jobs are those that result from activity in sectors that supply the materials or components used, but not exclusively so, by the renewables sector. | 11^th^ EurObserv’ER Report (EurOservER, 2011), 15^th^ EurObserv’ER Report (EurObservER 2015) |
| E2 | Change in socio-technical fit | Growth/decline in share of electricity for renewable energy sources in 2014 compared to 2010 in % | Share of renewable energy in gross electricity consumption in 2014. | 11^th^ EurObserv’ER Report (EurOservER, 2011), 15^th^ EurObserv’ER Report (EurObservER 2015) |
|  |  | Cross border capacity in 2010 as cross-border interconnections from and to a country and their conventional transmission capacity in MVA relative to the historic peak load in % | Cross-border capacity as of 31. December 2009, calculated from all cross-border interconnections from and to a country and their conventional transmission capacity in MVA relative to the historic peak load. For Sweden, this number might be slightly understated as transmission capacity is not reported for all connections. | ENTSO-E Characteristics of the ENTSO-E cross-frontier lines as of 31 December 2009 (ENTSO-E 2010) |
|  |  | Growth/decline wholesale price between 2010 and 2014 in Euro | Average annual electricity wholesale prices in 2010 compared to 2014. Prices have been adjusted for inflation and are expressed in Euro 2018 (constant price) terms. | Dashboard for energy prices in the EU and main trading partners (EU Commission 2022) |

**Data Analysis**

To determine the level of change (higher/lower) for the individual indicator, we compare individual countries to the average in our sample; “higher” signifies higher than the average in our sample. As shown below, we then categorize countries based on a set of predefined criteria as either having a low increase in STS maturity or a high increase (see Table B2).

| Low increase in maturity, countries meet the most criteria in this category:   - lower RE growth rate, - lower increase/decline in support, - lower decline LCOE and - lower increase in jobs. | High increase in maturity, countries meet the most criteria in this category:   - higher RE growth rate, - increase in levels of support - higher decline in LCOE and - higher increase in jobs. |
| --- | --- |

To analyze socio-technical fit of the new and incumbent energy systems, we follow the same process as for increase in STS maturity. We determine higher/lower fit relatively, by comparing the value of each socio-technical fit indicator to the average in our sample. Based on the criteria defined below, we classify countries as low and high fit (see Table B3).

| Low socio-technical fit, countries meet the most criteria in this category:   - higher share of RE in 2014, - lower cross-border transmission capacity and - higher decrease in the price of electricity. | High socio-technical fit, meets none or one of these criteria:   - lower share of RE 2014, - higher cross-border transmission capacity and - lower decrease/increase in the price of electricity. |
| --- | --- |

**Results STS properties**

**Table B2***:Comparative analysis of the RE STS property: technology maturity*

| **Country** | **Jobs percentage trend 2010-2014** | **Growth rate 2004-2014 (2004 index)** | **Support level trend 2010 and 2014 EUR/MWh** | **Average (PV/on-shore) LCOE trend 2010-2014 (USD/kWh)** | **Categorization** |
| --- | --- | --- | --- | --- | --- |
| France | -4% | 133% | +0.1 | -0.10 | Low increase |
| Germany | -3% | 298% | +4.4 | -0.1 | High increase |
| Italy | -24 | 207% | +9.9 | -0.13 | High increase |
| Spain | -38% | 199% | -0.17 | -0.09 | Low increase |
| Sweden | -8% | 123% | -0.16 | -0.01 | Low increase |
| United Kingdom | 210% | 515% | +1.74 | -0.14 | High increase |
| Average | 15%* | 246% | 2.63 | -0.094 |  |

*excluding the UK as an outlier

**Table B3***: Comparative analysis of the STS property: socio-technical fit*

| **Country** | **% share in 2014** | **Wholesale electricity price development 2010-2014 EUR/MWh** | **Transmission capacity relative to peak load** | **Categorization** |
| --- | --- | --- | --- | --- |
| France | 18% | -16 | 28% | High Fit |
| Germany | 28% | **-14** | 68% | High Fit |
| Italy | 37% | **-16** | 25% | Low fit |
| Spain | 40% | 4 | 24% | Low fit |
| Sweden | 62% | **-29** | 10% | Low fit |
| United Kingdom | 18% | +1 | **5%** | High fit |
| Average | 33.96 | -11.92 | 26.51% |  |

**References**

Cashore, B. and Howlett, M., 2007. Punctuating Which Equilibrium? Understanding Thermostatic Policy Dynamics in Pacific Northwest Forestry. *American Journal of Political Science*, 51 (3), 532–551.

CEER, 2011. *CEER Report on Renewable Energy Support in Europe*. Council of European Energy Regulators.

CEER, 2015. *Status Review of Renewable and Energy Efficiency Support Scheme in 2012 and 2013*. Council of European Energy Regulators.

ENTSO-E, 2010. *Statistical Yearbook 2009*.

EU Commission, 2022. *Dashboard for energy prices in the EU and main trading partners*.

EurObserv’ER, 2011. *The state of renewable energies in Europe. 11th EurObserv’ER report.*

EurObservER, 2015. *The State of Renewable Energies in Europe 15th EurObservER Report*.

IRENA, 2020. *Renewable Power Generation Costs in 2019*.

1. In the UK white papers are produced by the government and set out proposals for legislation. [↑](#footnote-ref-1)
2. Open-source software, available at: https://github.com/leifeld/dna. [↑](#footnote-ref-2)
